# A Comparative Evaluation of the Effect of Different Beverages on Colour Stability and Surface Micromorphology of Nanocomposite Restorative Material

**DOI:** 10.7759/cureus.41905

**Published:** 2023-07-14

**Authors:** Tulica Singh, Mahalakshmi V, Sasmita Sahu, Silpi Chatterjee, Asim Mustafa Khan, Maryam Faseeha Haqh, Vikas Singh

**Affiliations:** 1 Department of Pedodontics and Preventive Dentistry, Awadh Dental College and Hospital, Jamshedpur, IND; 2 Department of Dentistry, Bermo Sub-Divisional Hospital, Bermo, IND; 3 Department of Public Health Dentistry, Dr. D. Y. Patil Dental College & Hospital, Dr. D.Y. Patil Vidyapeeth (Deemed to be University), Pune, IND; 4 Department of Biomedical Dental Sciences, College of Dentistry, Imam Abdulrahman Bin Faisal University, Dammam, SAU; 5 Department of Pediatric Dentistry, Ram Dental Clinics, Dammam, SAU; 6 Department of Public Health Dentistry, Teerthanker Mahaveer Dental College & Research Centre, Moradabad, IND

**Keywords:** erosive potential, orange juice, coca-cola, carbonated drink, restorative materials, erosions, scanning electron microscope, artificial saliva, aerated drinks

## Abstract

Aim:This investigation was carried out to evaluate the color stability of a nanocomposite restorative material and the erosive potential of carbonated soft drinks (Coca-Cola; The Coca-Cola Company, Atlanta, Georgia, United States) and packaged orange juice (Real Fruit Power Orange; Dabur Ltd, Ghaziabad, Uttar Pradesh, India) on its surface micromorphology.

Materials and Methods: Sixty discs (2mm thick and 10mm diameter) of nanocomposite material (Herculite Précis; KaVo Kerr, Brea, California, United States) were prepared using a silicon cylindrical mold. Initially, all the specimens were stored in artificial saliva in five Petri dishes; 12 specimens in each dish. In the Petri dishes, the specimens were immersed in the respective beverages once or twice a day. Before and after each immersion, the specimens were stored in artificial saliva at room temperature. Artificial saliva was changed each day, i.e., every 24 hours. The whole procedure was carried out for three months and then evaluated for color stability using a spectrophotometer and surface micromorphology using a scanning electron microscope. Now, the exposure of specimens to aerated drinks (Coca-Cola) and packaged orange juice (Real Orange) was put to a halt, and specimens were kept continuously in artificial saliva. This procedure was carried out for one month and then evaluated for color stability. The information was analyzed using PASW Statistics for Windows, Version 18.0 (Released 2009; SPSS Inc., Chicago, United States). A p-value of 0.05 was considered significant.

Results: The p-value after three months, which is < 0.001 (p<0.05) indicates that the mean color difference values for groups I, II, III, IV, and V show a statistically significant change between the five groups, and similarly, the p-value after one month, which is < 0.001 (p<0.05) indicates that the mean color difference values for groups I, II, III, IV, and V show a statistically significant change between the five groups. Specimens immersed in the carbonated drink twice a day showed clinically more color change than packaged orange juice and artificial saliva on the composite restorative material. Coca-Cola, an aerated drink, was shown to have a higher erosive potential on the composite restorative material than Real Fruit Power Orange and fake saliva.

Conclusion: The findings are consistent with the hypothesis that repeated exposure to carbonated beverages (such as Coca-Cola and packaged juice) degrades the surface qualities of dental restorations.

## Introduction

Restorative dentistry is considered successful when it improves both the patient's oral health and appearance [1]. A smile's color, shape, and surface roughness all contribute to its unique appearance [1,2]. Composite resins are being used in dental clinics as one of the most common cosmetic restorative materials. The success of a restorative material, from an aesthetic standpoint, depends on how well it mimics the color and solidity of natural teeth [3].

Nanocomposite theory has recently been used in a way that has revolutionized the composite resin industry. Aesthetics, strength, and durability are three scientific concepts that modern nanocomposite material supply improves. The nanocomposite resin developed from advanced methacrylate resin meets the cosmetic and mechanical requirements for both anterior and posterior restoration [3,4]. In the past 20 years, nanocomposites with ingredients having a size smaller than 100 nm have rapidly developed. A flexible design of nanocomposites with hitherto unheard-of functions is made possible by the huge specific surface area and distinctive physicochemical characteristics of nanofillers. A hybrid material is not simply a physical amalgamation of many components, either. The resultant hybrid materials typically gain new properties after merging the multiscale components, and these qualities can be adjusted by the unique chemical and physical properties of individual components, structures, and interfaces between various components [2]. To what extent a resin matrix, photoinitiator, and inorganic filler contribute to intrinsic discoloration is a matter of quality. Adsorption and absorption of colorants in drinks and meals induce extrinsic discoloration as well as the duration of time that such substances are exposed [5]. Only when the composite resins are not fully polymerized or when they are exposed to food dyes or chemical dyes can the materials' original hues change noticeably. Since this is the most important factor influencing the color stability and long-term success of composite resins, these findings suggest that dental researchers and material scientists should concentrate on developing new resin-based materials with higher resistance to discoloration for use in aesthetic restorations [5].

Packaged juices and carbonated drinks may erode teeth, and their usage is especially high among children and teenagers. There is accumulating evidence that links the use of beverages to tooth deterioration [4]. The roughness of resin composites may affect the oral biofilm's adhesion, and bacterial accumulation is strongly reliant on the properties of the material surface. The potential for bacterial infection is influenced by the surface's physical and chemical characteristics [6]. Therefore, the capacity of a material to maintain its color is an important consideration for places where aesthetics are paramount [7]. Changes in particle size and the matrix/inorganic load ratio of the resins may be directly connected to the color stability of the finished product [7]. Color stability and surface micromorphology of restorative materials used in pediatric dentistry are largely unexplored. Given aesthetics are now of the utmost importance for parents as well as their children, these are very important [8,9].

Thus, the research question was whether the repeated exposure to carbonated soft drinks and packaged orange juice degrade the color stability and surface micromorphology of a nanocomposite restorative material.

## Materials and methods

This study was conducted at Manav Rachna Dental College, Faridabad, India. There was a collaboration with the Central Research Facility at the Indian School of Mines in Dhanbad, Jharkhand, India for scanning electron microscopy (SEM) and with the Department of Textile Technology at the Indian Institute of Technology, New Delhi; India, for use of the spectrophotometer.

Specimen preparation

Using a silicon cylindrical mold, 60 discs of nanocomposite material (Herculite Précis, A2 shade; Envista Holdings Corporation, Brea, California, United States) were created. The formula used for sample size calculation was: n = (Zα/2 + Zβ)^2 * (2 * σ^2) / Δ^2, where:n is the required sample size per group, Zα/2 is the critical value for a two-tailed test at a desired significance level (α), Zβ is the critical value for the desired power (1-β), which is typically set at 80% or 90%, σ^2 is the estimated population variance and Δ is the minimum detectable difference between groups that you want to detect.

Each disc measured 10 mm in diameter and 2 mm in thickness. The substance was poured into the mold after pressing a polyester strip onto the mold's exterior using a glass plate to prevent the development of bubbles. Light-emitting diode (LED) devices (Ivoclar, Schaan, Liechtenstein) were placed at a distance of 1 mm from each specimen and used to cure the discs for 40 seconds, 600mW/cm^2^ from both the top and bottom. After the resin had been set, the specimen was taken out of the mold and polished for 60 seconds with wet 600- and 1200-grit silicon carbide paper [10]. We used a digital caliper (SDN 10;Baker Gauges India Private Limited, Pune, Maharashtra, India) to gauge the thickness of the discs. Once everything was prepped, we let the specimens sit undisturbed for 30 minutes before doing the thermocycling test. In order to imitate aging, the specimens were heated and cooled 1500 times between 5 degrees Celsius and 50 degrees Celsius, with a 30-second dwell period in each water bath [6]. The pH of different solutions was verified by a pH meter (pHep® Waterproof Pocket pH Tester; Hanna Instrument Ltd, Bedfordshire, United Kingdom), which indicated no changes during the period of treatment.

Five Petri dishes were taken with 12 specimens in each, and initially, all the specimens were stored in artificial saliva. The specimens in each Petri dish were also immersed in the respective beverages according to the groups. There were thus five groups with the following pattern: (i) Group I: Specimens were stored in artificial saliva without being exposed to any beverages and were taken as the control group; (ii) Group II: Specimens were exposed to an aerated drink (Coca-Cola; The Coca-Cola Company, Atlanta, Georgia, United States) once a day for a time period of 30 minutes, after which they were returned to artificial saliva; (iii) Group III: Specimens were exposed to an aerated drink (Coca-Cola) twice a day, each lasting for 30 minutes, evenly distributed over a 12-hour period, and after each immersion, they were returned to artificial saliva; (iv) Group IV: Specimens were exposed to packaged orange juice (Real Fruit Power Orange; Dabur Ltd, Ghaziabad, Uttar Pradesh, India) once a day for a period of 30 minutes, and after immersion, they were returned to artificial saliva; (v) Group V: Specimens were exposed to packaged orange juice (Real Fruit Power Orange) twice a day, each lasting for 30 minutes, evenly distributed over a 12-hour period, and after each immersion, they were returned to artificial saliva [8].

Before and after each immersion, the specimens were stored in artificial saliva (not exposed to any beverage) at room temperature. Artificial saliva was changed each day, i.e., every 24 hours. The whole procedure was carried out for three months and then evaluated for color stability using a spectrophotometer (Gretag Macbeth^TM^, Color-Eye® 7000A; X-Rite, Inc., Grand Rapids, Michigan, United States) and surface micromorphology using an SEM (Zeiss Supra 55-VP FE-SEM; Carl Zeiss AG, Oberkochen, Baden-Württemberg, Germany).

**Figure 1:**
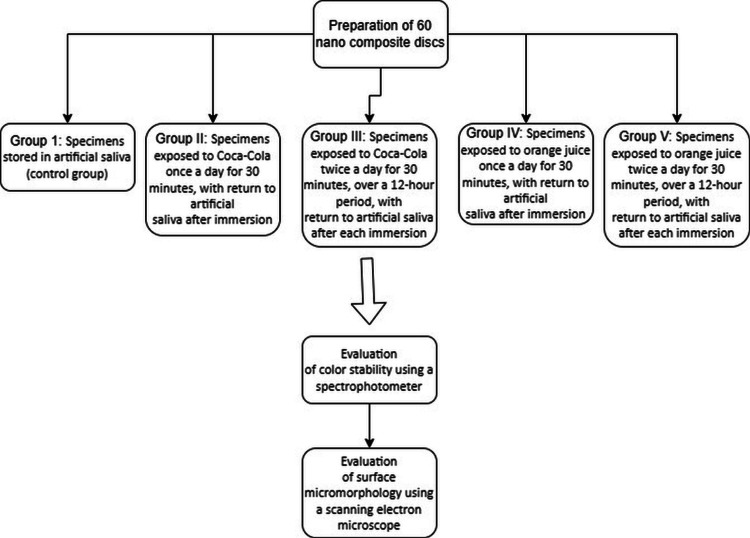
Flow chart for the study

The process is detailed in Figure 1.

Phase II of the experiment

The exposure of specimens to aerated drinks (Coca-Cola) and packaged orange juice (Real Fruit Power Orange) was put to a halt, and specimens were kept continuously in artificial saliva. Artificial saliva was changed each day, i.e., every 24 hours. The whole procedure was carried out for one month and then evaluated for color stability using a spectrophotometer (Gretag Macbeth Color-Eye® 7000A) and surface micromorphology using an SEM (Zeiss Supra 55-VP FE-SEM).

Color stability analysis

The spectrophotometer measures color differences in three dimensions using the Commission Internationale de l'Eclairage (CIEL*a*b* color space system. The L* indicates the brightness or darkness of the image. The a* indicates how much of a color is red (positive a*) or green (negative a*). Yellowness (a positive b* value) and blueness (a negative b* value) are quantified by the b* value. When comparing data between groups, we used the formula E* = [(L*)2 + (a*)2+ (b*)2]1/2 to get the color difference (E). The active point of the spectrophotometer was positioned in the middle of each specimen for all three measurements; the device then averaged these results for use in the final statistical analysis.

Surface micromorphology analysis

**Figure 2:**
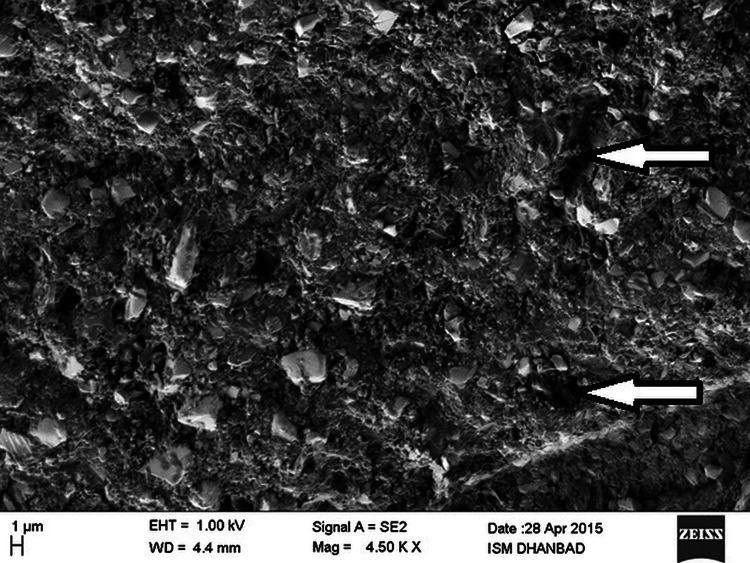
SEM photograph of surface to see the erosions Credit: SEM was done at the Central Research Facility at the Indian School of Mines in Dhanbad, Jharkhand, India, using the SUPRA 55VP FE-SEM (Carl Zeiss AG, Oberkochen, Baden-Württemberg, Germany) SEM: scanning electron microscopy

The effect of each storage agent at different frequencies on the surface micromorphology of the material in each group was determined at the end of three months and one month, respectively, using the Supra 55 FE-SEM. The interaction between the electron beam and the specimen generates signals that create detailed images of the surface topography at a microscopic level (Figure 2).

Data analysis

The data was analyzed using PASW Statistics for Windows, Version 18.0 (Released 2009; SPSS Inc., Chicago, United States). A one-way ANOVA with post hoc Tukey's Honest Significant Difference (HSD) test was used for normal data to compare means across more than two groups. The paired t-test was performed to examine the significance of differences between means at two separate times. The significance threshold (value) was established at 0.05. 

## Results

The intergroup comparison of color differences at the end of the first three months and the final one month is given in Table 1. In the case of mean values of color difference (∆E) after three months, statistical analysis showed that Group I was significantly different from Groups II, III, IV, and V (p < 0.001). Similarly, there was a statistically significant difference (p < 0.001) between Group II and Groups III, IV, and V. It was also determined that there was a statistically significant difference (p < 0.001) between Group III and Groups IV and V, and that there was a statistically significant difference (p < 0.001) between Group IV and Group V (Table 2). In the case of mean values of ∆E after the final one month, Group I was shown to be statistically distinct from Groups II, III, IV, and V (p < 0.001). Group II was significantly different from Groups III and V (p <0.001), but not from Group IV (p>0.001). Significant differences were identified between Group IV and Group V (p < 0.001) and between Group III and Group V (p < 0.001) (Table 2).

**Table 1:** Intergroup comparison of color difference (∆E) values using one-way ANOVA

Variables		Sum of squares	df	Mean square	F	p-value
Values of color difference (∆E), three months	Between Groups	120.196	4	30.049	523.164	<0.001
	Within Groups	3.159	55	0.057		
	Total	123.355	59			
Values of color difference (∆E), final one month	Between Groups	64.381	4	16.095	346.101	<0.001
	Within Groups	2.558	55	0.047		
	Total	66.939	59			

**Table 2:** Intergroup comparison of color difference after application of post hoc Tukey test

(I) Group	(J) Group	Values of color difference (∆ E), three months	Values of color difference (∆E), final one month
		p-value	p-value
				Artificial Saliva	Coca-cola once a day	<0.001	<0.001
Coca-cola twice a day	<0.001	<0.001		
Orange Juice once a day	<0.001	<0.001		
Orange juice twice a day	<0.001	<0.001		
Coca-cola once a day	Coca-cola twice a day	<0.001	<0.001
	Orange Juice once a day	<0.001	0.057
	Orange juice twice a day	<0.001	<0.001
Coca-cola twice a day	Orange Juice once a day	<0.001	<0.001
	Orange juice twice a day	<0.001	<0.001
Orange Juice once a day	Orange juice twice a day	<0.001	<0.001

Therefore, there is a statistically significant difference between the mean color difference values for groups I, II, III, IV, and V after three months, as indicated by the p-value of 0.001 (p < 0.05), and there is a statistically significant difference after one month, as indicated by the p-value of 0.001 (p < 0.05) (Table 3).

**Table 3:** Intergroup comparison of color difference (∆E) values after application of paired t-test

Groups		N	Mean	Standard deviation	t-value	p-value
Group I	Values of color difference( ∆E) 3m	12	1.06	0.113	5.640	<0.001
	Values of color difference (∆ E) 1m	12	1.30	0.087		
Group II	Values of color difference(∆E) 3m	12	2.71	0.330	5.757	<0.001
	Values of color difference (∆E) 1m	12	1.79	0.293		
Group III	Values of color difference(∆E) 3m	12	5.37	0.205	123.350	<0.001
	Values of color difference (∆E) 1m	12	4.28	0.221		
Group IV	Values of color difference(∆E) 3m	12	2.28	0.237	11.53	<0.001
	Values of color difference (∆ E) 1m	12	2.03	0.222		
Group V	Values of color difference( ∆E) 3m	12	3.24	0.260	7.176	<0.001
	Values of color difference (∆ E) 1m	12	2.74	0.202		
Overall	Values of color difference(∆ E) 3m	60	2.93	1.446	7.08	<0.001
	Values of color difference ( ∆E) 1m	60	2.43	1.065		

SEM photomicrographs of the nanohybrid resin composite were taken at a magnification of 4.50 KX and a working distance range of 3-4.5 mm. Specimens stored in Coca-Cola and Real Fruit Power Orange at different frequencies showed loss of integrity, large voids, and missing filler particles, and consequently greater surface roughness after three months and one month, when compared with control groups. All five groups, when stored in artificial saliva continuously for the final one month, showed less erosiveness as compared to specimens of the five groups when exposed to different beverages at different frequencies after three months, except the control group, which showed more erosiveness after the final one month in contrast to its erosion after three months.

## Discussion

The present study was carried out to evaluate the color stability of a nanocomposite restorative material when exposed to aerated drinks (Coca-Cola) and packaged orange juice (Real Fruit Power Orange) at different frequencies and to investigate the erosive potential of these acidic agents on the surface micromorphology of the restorative material. Artificial saliva was used as a control.

Coca-Cola is a popular soft drink with the lowest pH (3.8) among the beverages used in the present study and is made mostly of phosphoric acid, which has a preservation quality and a distinct sour flavor. Citrus fruits contain several different types of acids, including citric, malic, tartaric, benzoic, oxalic, and succinic acids. Citric acid, a weak tricarboxylic acid composed of 2 hydroxyl 1,2,3-propane, is the primary organic acid in citric juices. Since packaged orange juice is readily available and has an acidity that is considered to be on par with that of soft drinks, it was chosen for this investigation. The color change was compared using three values of ∆E, namely, the overall color change after a period of immersion, similar to the research of Bagheri et al. [10]. Because of its advantages, including repeatability, sensitivity, and objectivity, this approach was selected to measure ∆E. The larger the value of ∆E, the larger the gap between the two samples. In dentistry, an ∆E value of 3.3 or below is considered safe for human use [11]. It was seen that when nanocomposite resin was exposed to immersion media Coca-Cola (twice a day), it showed the greatest color difference as compared to other groups after three months and the final one month, but when nanocomposite resin composite was exposed to Real Fruit Power Orange, it showed that the color difference of the specimens in orange juice exposed twice a day in the final one month of immersion was greater than the color difference of the specimens in carbonated drink exposed once a day and packaged orange juice exposed once a day after three months of immersion. The type of organic matrix, the size and distribution of the filler particles, and the exposure of the material to low-PH foods, beverages, and mouthwash solutions are all factors that affect composite parameters like color stability and surface roughness [11].

After three months of exposure to various immersion media at various frequencies, there was a noticeable color difference in all the groups. However, when the exposure to the various immersion media was stopped after a month and the specimens were continuously kept in artificial saliva, the color difference stopped and was only slightly reduced in all groups other than the control group. According to our research, dipping samples into artificial saliva caused a slight increase in color shift (E: 1.30). These findings were also noticed by Domingos et al. [12]. The test samples become discolored as a result of monomers like diglycidyl methacrylate, urethane dimethacrylate, and triethylene glycol dimethacrylate. This results in internal color changes because the resin matrix turns hydrophilic, dramatically increasing its absorption of water and other fluids [10]. Two additional potential causes of color shifts are filler particle dispersion and photoinitiator systems [13].

In the present study, a single composite restorative material was selected to examine the impact of various drinks on the composite material at various frequencies. Hotwani et al. investigated color stability by immersing two hybrid tooth-colored materials (resin-modified glass ionomer cement (RMGIC) and giomer) in a variety of children's drinks (orange juice, Bournvita (Cadbury, Uxbridge, United Kingdom) milk, and soda) [14]. The results showed that the color stability of giomer specimens was superior to that of RMGIC specimens. The most dramatic color changes occurred after four weeks of soda consumption. Salman et al. studied the effects of three commonly consumed drinks (coffee, tea, and carbonated drink) on the 24-hour, seven-day, and 30-day color retention of acrylic denture teeth [15]. The results showed that carbonated drinks caused the most dramatic shift in color.

On the contrary, the results were not consistent with the findings reported by Erta et al. who compared the discoloration of a posterior composite resin restorative material to that of water, tea, carbonated drink, coffee, red wine, and alcohol on two nanohybrids, two micro hybrids, and a control group [13]. Water always had the lowest E value across all materials tested, whereas red wine had the highest. The effect of different lighting conditions and immersion mediums on the color retention of a nano-filled composite resin was studied by Domingos et al. [12]. Incubation of the specimens in the four media (coffee, tea, Coca-Cola, and fake saliva) lasted for 60 days. The composite resin's color stability was most affected by coffee.

In the present study, SEM photomicrographs of the nanohybrid resin composite were taken in different beverages. Employing the frequency-dependent interrupted specimen immersion method, we examined the effects of carbonated beverage (Coca-Cola) and packaged fruit juice (Real Fruit Power Orange) exposure. Because phosphoric acid is more erosive than other organic hydroxy acids like citric, malic, and lactic acids at the same pH and concentration [16], specimens treated with Coca-Cola twice daily had the roughest surface. The reason for the higher wear rate of composites may be due to the acid attack on the resin, which causes softening of bisphenol-A-glycidyl methacrylate (Bis GMA)-based polymers and leaching of triethylene glycol dimethacyrlate [17]. The results of the present study are in accordance with a study conducted by Rajavardhan et al., which has shown Coca-Cola to be the most erosive [18]. However, these results did not jibe with those published by Bamise et al., who determined that fruit juices had a higher erosive potential than carbonated drinks [19]. The results of the present study also varied from the findings of Meurman and Ten Gate, who reported citric acid to be more erosive than phosphoric acid [20]. Hence, the danger is the frequent use of soft drinks and fruit juices over time. The frequency of exposure to beverages can be concluded to be one of the critical factors affecting color stability and surface micromorphology.

The study has some limitations. The study had a relatively small sample size, limiting the generalizability of the findings. The evaluation period was relatively short, and a longer duration would provide a better understanding of long-term effects. The focus on specific beverages limits the ability to draw comparisons. The use of artificial saliva as a storage medium may not fully replicate the oral environment, potentially influencing the outcomes. Other factors such as patient-related factors and different restorative materials were not considered, limiting the overall scope and applicability of the study. Further studies on different drinks are needed to fully understand how beverage consumption habits affect the surface qualities of composite resins and the durability of restorations over time.

## Conclusions

The study findings demonstrate that repeated exposure to carbonated soft drinks, specifically Coca-Cola, can lead to significant color change and surface degradation of a nanocomposite restorative material. After three months of immersion, specimens exposed to Coca-Cola twice a day exhibited the highest degree of color change compared to those exposed to packaged orange juice and artificial saliva. Furthermore, the erosive potential of Coca-Cola was found to be greater than that of Real Fruit Power Orange and artificial saliva, indicating its detrimental effect on the surface micromorphology of the composite restorative material. These results support the hypothesis that regular consumption of carbonated beverages can negatively impact the longevity and aesthetic qualities of dental restorations.

## References

[1] Sikri VK (2010). Color: Implications in dentistry. J Conserv Dent.

[2] Samra AP, Pereira SK, Delgado LC, Borges CP (2008). Color stability evaluation of aesthetic restorative materials. Braz Oral Res.

[3] Fontes ST, Fernández MR, de Moura CM, Meireles SS (2009). Color stability of a nanofill composite: effect of different immersion media. J Appl Oral Sci.

[4] Khatri A, Nandlal B (2010). Staining of a conventional and a nanofilled composite resin exposed in vitro to liquid ingested by children. Int J Clin Pediatr Dent.

[5] Ren YF, Feng L, Serban D, Malmstrom HS (2012). Effects of common beverage colorants on color stability of dental composite resins: the utility of a thermocycling stain challenge model in vitro. J Dent.

[6] Poggio C, Dagna A, Chiesa M, Colombo M, Scribante A (2012). Surface roughness of flowable resin composites eroded by acidic and alcoholic drinks. J Conserv Dent.

[7] Gupta G, Gupta T (2011). Evaluation of the effect of various beverages and food material on the color stability of provisional materials - An in vitro study. J Conserv Dent.

[8] Tunc ES, Bayrak S, Guler AU, Tuloglu N (2009). The effects of children's drinks on the color stability of various restorative materials. J Clin Pediatr Dent.

[9] Shivaughn MM, William AJ, Quinta MM, Marta EO (2014). A nanohybrid investigated: the correlation between roughness parameters and color after instrumentation. Int J Clin Pediatr Dent [discontinued].

[10] Bagheri R, Burrow MF, Tyas MJ (2007). Surface characteristics of aesthetic restorative materials - an SEM study. J Oral Rehabil.

[11] Kalita T, Kalita C, Das L (2023). Comparative evaluation of colour stability and surface roughness of nanohybrid composite resins in mouth rinse and colouring beverages. Cureus.

[12] Domingos PA, Garcia PP, Oliveira AL, Palma-Dibb RG (2011). Composite resin color stability: influence of light sources and immersion media. J Appl Oral Sci.

[13] Ertaş E, Güler AU, Yücel AC, Köprülü H, Güler E (2006). Color stability of resin composites after immersion in different drinks. Dent Mater J.

[14] Hotwani K, Thosar N, Baliga S (2014). Comparative in vitro assessment of color stability of hybrid esthetic restorative materials against various children's beverages. J Conserv Dent.

[15] Salman FD, Al-Gaban RM (2011). The effect of various staining agents on color stability of acrylic denture teeth materials. (In vitro study). J Kerbala Univ.

[16] Guler AU, Yilmaz F, Kulunk T, Guler E, Kurt S (2005). Effects of different drinks on stainability of resin composite provisional restorative materials. J Prosthet Dent.

[17] West NX, Hughes JA, Addy M (2001). The effect of pH on the erosion of dentine and enamel by dietary acids in vitro. J Oral Rehabil.

[18] Rajavardhan K, Sankar A, Kumar M, Kumar K, Pranitha K, Kishore K (2014). Erosive potential of cola and orange fruit juice on tooth colored restorative materials. Ann Med Health Sci Res.

[19] Bamise CT, Ogunbodede EO, Olusile AO, Esan TA (2007). Erosive potential of soft drinks in Nigeria. World J Med Sci.

[20] Meurman JH, ten Cate JM (1996). Pathogenesis and modifying factors of dental erosion. Eur J Oral Sci.

